# Supplementary material for: Quarantine supervision of Wood Packaging Materials (WPM) at Chinese ports of entry from 2003 to 2016
Source: PLoS One. 2021 Aug 5;16(8):e0255762. doi: 10.1371/journal.pone.0255762 (PMC8341634; doi:10.1371/journal.pone.0255762)
Supplement: S1 Table — (DOCX) [file pone.0255762.s001.docx]

S1 Table Definitions of terms

|  | Definition | Notes |
| --- | --- | --- |
| Pests | Organisms that are harmful to plants or plant products; these may include plants, animals, pathogens, etc. The records in the interception data used in this paper are all for pests. Examples include *Sinoxylon* spp. (non-Chinese), *Platypus parallelus*, saprophytic nematodes, *Bursaphelenchus doui*, Araneidae, Acaridae, etc. | 1. This is especially common when insects are collected as larvae. Because the immature stages of many insects cannot be identified to the species level and time constraints do not allow rearing insects to the adult stage, insects may be identified only to the order, family, or genus level in these cases[1, 2].  2. When quarantine customs officers found vector insects (*Monochamus alternatus*, etc.) or signs of blue staining on the wooden pallet, they immediately extracted wooden pallet samples and sent them to the laboratory to determine whether nematodes were present. |
| Living pests | Pests with vital signs at the time of interception |  |
| Dead pests | Pests without vital signs at the time of interception |  |
| Quarantine pests | Pests listed on the Catalogue of Quarantine Pests for Import Plants to the PRC. For example, *Heterobostrychus aequalis*, *Xyleborus* spp. (non-Chinese), *Bursaphelenchus xylophilus*, *Platypus parallelus, Xanthium spinosum*, *Monochamus* spp. (non-Chinese), *Sirex noctilio*, etc*.* |  |
| Non-quarantine pests | Pests not listed on the Catalogue of Quarantine Pests for Import Plants to the PRC. For example, *Apriona germari*, *Anoplophora chinensis*, *Lycaeopsis zamboangae*, *Hylurgus ligniperda*, *Bursaphelenchus mucronatus*, etc. |  |

**References**

1. Wu YK, Trepanowski NF, Molongoski JJ, Reagel PF, Lingafelter SW, Nadel H, et al. Identification of wood-boring beetles (Cerambycidae and Buprestidae) intercepted in trade-associated solid wood packaging material using DNA barcoding and morphology. Sci Rep. 2017;7:40316.

2. Haack RA. Intercepted Scolytidae (Coleoptera) at U.S. ports of Entry: 1985–2000. Integr Pest Manag Rev. 2001;6(3-4):253-82.
