# Supplementary material for: Quarantine supervision of Wood Packaging Materials (WPM) at Chinese ports of entry from 2003 to 2016
Source: PLoS One. 2021 Aug 5;16(8):e0255762. doi: 10.1371/journal.pone.0255762 (PMC8341634; doi:10.1371/journal.pone.0255762)
Supplement: S2 Table — (DOCX) [file pone.0255762.s002.docx]

S2 Table Matrix Eigenvalue and Cumulative Variance Contribution Rate of Factor Analysis

| factor | eigenvalue | variance contribution rate % | Cumulative contribution rate % |
| --- | --- | --- | --- |
| 1  2 | 4.183 | 69.71 | 69.71 |
|  | 1.236 | 20.60 | 90.31 |
