# Supplementary material for: Quarantine supervision of Wood Packaging Materials (WPM) at Chinese ports of entry from 2003 to 2016
Source: PLoS One. 2021 Aug 5;16(8):e0255762. doi: 10.1371/journal.pone.0255762 (PMC8341634; doi:10.1371/journal.pone.0255762)
Supplement: S3 Table — (DOCX) [file pone.0255762.s003.docx]

S3 Table Rotated factor loading matrix

| variable | factor 1 | factor 2 |
| --- | --- | --- |
| Nematode | 0.907 | 0.137 |
| storage pests | 0.898 | 0.197 |
| Cerambycidae | 0.860 | 0.451 |
| Platypodidae | 0.206 | 0.945 |
| Bostrichidae | 0.155 | 0.941 |
| Scolytidae | 0.557 | 0.796 |
