# Supplementary material for: Quarantine supervision of Wood Packaging Materials (WPM) at Chinese ports of entry from 2003 to 2016
Source: PLoS One. 2021 Aug 5;16(8):e0255762. doi: 10.1371/journal.pone.0255762 (PMC8341634; doi:10.1371/journal.pone.0255762)
Supplement: S4 Table — (DOCX) [file pone.0255762.s004.docx]

S4 Table The factor scores and rankings of original countries (regions) of intercepted entry WPM pests

| Country (Region) | FAC_1 | FAC_2 | synthesis score | Rank |
| --- | --- | --- | --- | --- |
| Germany | 7.76128 | -1.13653 | 5.176263 | 1 |
| United States | 5.3759 | -0.08432 | 3.73017 | 2 |
| Taiwan(China) | 3.07806 | 7.64298 | 3.72017 | 3 |
| South Korea | 5.63648 | -1.27746 | 3.666033 | 4 |
| Japan | 2.18316 | 0.04471 | 1.531091 | 5 |
| Hong Kong(China) | 1.43014 | 1.59038 | 1.324569 | 6 |
| Italy | 1.80593 | -0.32793 | 1.19136 | 7 |
| France | 1.33677 | -0.46882 | 0.835285 | 8 |
| Singapore | -0.23462 | 3.80325 | 0.619916 | 9 |
| Brazil | 0.58505 | 0.6621 | 0.544231 | 10 |
| Russia | 0.74813 | -0.11128 | 0.498598 | 11 |
| Indonesia | -0.30372 | 3.35157 | 0.4787 | 12 |
| Chile | 0.83332 | -0.49762 | 0.478398 | 13 |
| Belgium | 0.737 | -0.35049 | 0.441562 | 14 |
| Malaysia | -1.09227 | 5.70362 | 0.413524 | 15 |
| United Kingdom | 0.63022 | -0.2525 | 0.387311 | 16 |
| Netherlands | 0.5682 | -0.26658 | 0.341177 | 17 |
| Thailand | -0.84547 | 4.49279 | 0.336138 | 18 |
| Spain | 0.55095 | -0.24975 | 0.332619 | 19 |
| India | -0.38769 | 2.50169 | 0.245089 | 20 |
| Turkey | 0.30767 | -0.22737 | 0.167639 | 21 |
| Sweden | 0.19926 | -0.261 | 0.085138 | 22 |
| Canada | 0.15826 | -0.14126 | 0.081223 | 23 |
| Mexico | 0.12855 | -0.13163 | 0.062496 | 24 |
| Czech Republic | 0.08735 | -0.21574 | 0.016449 | 25 |
| Argentina | -0.01822 | 0.07776 | 0.003317 | 26 |
| Austria | 0.07225 | -0.25116 | -0.00137 | 27 |
| Poland | 0.06928 | -0.24415 | -0.002 | 28 |
| Belarus | 0.04638 | -0.19449 | -0.00773 | 29 |
| Ukraine | 0.02511 | -0.21608 | -0.02701 | 30 |
| Philippines | -0.24341 | 0.68895 | -0.02776 | 31 |
| Switzerland | 0.01486 | -0.21691 | -0.03432 | 32 |
| Vietnam | -0.22266 | 0.57375 | -0.03702 | 33 |
| Australia | -0.01634 | -0.17741 | -0.04794 | 34 |
| New Zealand | -0.06838 | -0.14371 | -0.07727 | 35 |
