# Supplementary material for: Quarantine supervision of Wood Packaging Materials (WPM) at Chinese ports of entry from 2003 to 2016
Source: PLoS One. 2021 Aug 5;16(8):e0255762. doi: 10.1371/journal.pone.0255762 (PMC8341634; doi:10.1371/journal.pone.0255762)
Supplement: Attachment — Submitted filename: Respnse to Reviewers.docx [file pone.0255762.s008.docx]

Dear Editor,

On behalf of my co-authors, we thank you very much for giving us an opportunity to revise our manuscript, we appreciate editor and reviewers very much for their positive and constructive comments and suggestions on our manuscript entitled “**Quarantine Supervision of Wood Packaging Materials (WPM) at Chinese Ports of Entry: 2003–2016**”. We have studied reviewers’ and Editor's comments carefully and have made revisions marked in highlight. Attached please find the revised version, which we would like to submit for your kind consideration.

We tried our best to improve the manuscript and made some changes in the manuscript. These changes will not affect the content and framework of the paper. We hope that our revision could address all of the questions by the reviewers and Editor.

Thank you very much for your considering our manuscript for publication. We are looking forward to hearing from you soon.

Prof. Juan Shi

Beijing Key Laboratory of Forest Pest Control, Forestry College, Beijing Forestry University

P.O. Box 113, 35 Qinghua East Road, Haidian District, Beijing 100083, P. R. China

Tel: +86-10-62336423

Fax: +86-10-62336423

E-mail: [shi_juan@263.net](mailto:shi_juan@263.net)

-----------------------------------------------------------------------------------------------------------------

**The following is a point-to-point response to Editor , reviewer 1, reviewer 2 and reviewer 3’s comments.**

-----------------------------------------------------------------------------------------------------------------

**Response to Editor:**

*Please ensure that your manuscript meets PLOS ONE's style requirements, including those for file naming.*

**Answer**:

Thank you very much for your comments. We have found The PLOS ONE style templates and modified the article according to its requirements

*Please ensure that you include a title page within your main document.*

**Answer**:

Thank you for editor's advice, We have merged the title page and the manuscript file.

*We note that you have indicated that data from this study are available upon request. PLOS only allows data to be available upon request if there are legal or ethical restrictions on sharing data publicly.*

**Answer**:

There are ethical or legal restrictions on sharing a de-identified data set，The data, from the “Animal and Plant Quarantine Information Resource Sharing Service Platform” (http://www.customs.gov.cn/),data contain potentially sensitive information, due to the complexity of the database, possible misunderstandings, and even international trade disputes, these data are rarely published. The data presented in this study are available on request from the corresponding author（[shi_juan@263.net](mailto:shi_juan@263.net)）.

The final dataset has been uploaded to Qualitative Data Repository (QDR) (doi:10.5064/F6KSSLAH),but the status "Unpublished" is displayed. I can't operate at the moment, I uploaded the data together with Manuscript, I hope the editor can give me help, thanks

*We note that* *Figure 6 in your submission contain map images which may be copyrighted.*

**Answer**:

Thank you for your comments on our manuscript. The map in Figure 6 is from a Chinese public website(<http://bzdt.ch.mnr.gov.cn/index.html>), which is allowed to publish the content specifically under the CC BY 4.0 license.

**Response to Reviewer 1:**

*If you have the data, looking at different species of pests (insects, mites, etc,….) might be a beneficial addition to the paper.*

**Answer**:

Thank you for your comments on our manuscript. in our Paper, the purpose is to used a variety of statistical approaches to demonstrate how intercepted pests differ among countries of origin and provincial ports, We used 14 years of 464,512 wood packaging interception records to analyze the four aspects of goods imported, Content of origin, Country of Origin, and Inspection stations. We analyzed the insects of Cerambycidae, Scolytidae, Platypodidae, and Bostrichidae using cluster analysis, factorial and correspondence analyses. It would be very interesting to focus on new and different species of pests, which I can study in more detail in the next papers.

*I suggest to the authors to report the geographic coordinates of the study areas.*

**Answer**:

We completely agree with the reviewer. Those comments are very helpful for revising and improving our paper. We have made Figure 6 according to above suggestions to present geographic coordinates information.

*figure 7: Explain which type of pesticides were used, how the number of treatments required and the choice of pesticides are established. I suggest authors to motivate the choice of pesticides used in the study. On the basis of what they were chosen? Are they the ones most used by companies? Are there any relationships between them? Please improve this aspect.*

**Answer**:

Thank you for your comments on our manuscript. Figure 7 mainly illustrates the different ports in China in the face of different quarantine status different survival status of pests to deal with measures (destruction or not), for the treatment of the type of pesticide used and related issues, in the paper is not relevant, if necessary we can added it to the annex.

*How are the pests ( insects, mites, nematodes, etc,…) sampled? Identification is done by which method? Please provide this information.*

**Answer**:

Your suggestion is greatly appreciated. I have explained it in annex, please look at the tableS1

**Response to Reviewer 2:**

**Introduction,**

*line 51: Please, add the Authority of the specie as follows: “Dendroctonus valens LeConte and Bursaphelenchus xylophilus (Steiner & Bührer) Nickle”*

**Answer**: Revised accordingly.

*Line 66: Please, change the line as follows: "analysis of Coleoptera in WPM pest interception data from"*

**Answer**: Revised accordingly.

*Line 72: Please, change the line as follows: "Scolytidae, Cerambycidae, and two other families of insects"*

**Answer**: Revised accordingly.

*Line 86: Please, put a space after "strategies"*

**Answer**: Revised accordingly.

*Line 87: 2003 or 2004? In other parts of the paper and in the title there is 2004: please, check and correct*

**Answer**: Thank you for your careful work. It is 2003, we have rechecked the other parts of the paper and made revisions to confirm.

**MATERIALS AND METHODS**

*Line 118: 2003 or 2004?*

**Answer**: Thank you for your careful work. It is 2003, we have made revisions.

*Line 122: Please, put a space after "records"*

**Answer**: Revised accordingly.

*Line 142: Please, correct the table by adding a line to separate each category of pests from other. In the current way it is difficult to read correctly. In addition, please, put a space between "cases" and "[16, 28]"*

**Answer**: Thank you for your comments on our manuscript. Those comments are very helpful for revising and improving our paper.We have modified the table and added it to the annex

**RESULTS AND DISCUSSION**

*Line 151: Please, put a space between "officers" and "[29-31]"*

**Answer**: Revised accordingly.

*Lines 155-156: Please, put a space between "interceptions" and "[32-33]"*

**Answer**: Revised accordingly.

*Lines 195-198: The Fig. 3 not clearly explains the results: the graph does not show the trend in the four quarters in each year as said in the text*

**Answer**: Thank you very much for your comments. The scale of the horizontal coordinates in Figure 3 is not clearly explained, and each scale represents a quarter rather than a year. We have modified the Figure so that it clearly explains the results.

*Line 200: Please, put a space between "sectors" and "[35-36]"*

**Answer**: Revised accordingly.

*Line 210: Please, put a space between "itself" and "[37]"*

**Answer**: Revised accordingly.

*Line 253: Please, put a space between "underestimated" and "[38]"*

**Answer**: Revised accordingly.

*Line 255: Table 2: I suggest of enlarge the second column (intitled "Rank order by total interceptios…" ) and fit the text in order to make the table more readible*

**Answer**: Your suggestion is greatly appreciated. We have revised the Table 2.

*Line 273: Please, put a space between "categories" and "(Table S2)"*

**Answer**: Revised accordingly.

*Line 279: Please, remove brachet after "scores"*

**Answer**: Revised accordingly.

*Line 280: Please, remove "(xylophagous insects)" because is redundant (already said in line 276)*

**Answer**: Revised accordingly.

*Line 297: Please, put a space between "species" and "[39, 40]"*

**Answer**: Revised accordingly.

*Line 300: Please, put a space between "damage" and "[41, 42]"*

**Answer**: Revised accordingly.

*Line 302: Please, put a space between "(regions)" and "[43, 44]"*

**Answer**: Revised accordingly.

*Line 305: Fig 5: Please, improve the readability of the figure*

**Answer**: Revised accordingly.

**INSPECTION STATIONS**

*Line 314: Please, put a space between "that" and "-"*

**Answer**: Revised accordingly.

*Line 315: Please, put a space between "recorded" and "-"*

**Answer**: Revised accordingly.

*Line 330: Please, put a space between "provinces" and "[45]"*

**Answer**: Revised accordingly.

*Line 346-350: In the Fig 6 there are not the abbreviations that are indicated in the caption. Olease, check and correct*

**Answer**: Thank you for your careful work. “CQ” is an abbreviation of "Chongqing".We have modified Figure 6.

*Line 387: Please, put a space between "cargo" and "[46]"*

**Answer**: Revised accordingly.

**REFERENCES**

*Line 476: Please, change "126+8" in "126-8"*

**Answer**: Revised accordingly.

*Line 560: Please, change "Sci．. 2013" in "Sci. 2013"*

**Answer**: Revised accordingly.

**Response to Reviewer 3:**

**Introduction**

*Line 64: to move to discussion*

**Answer:** Thank you for your comments on our manuscript. We refer to other literature, and we think this part can be put in Introduction, our discussion is mainly for the results, so we feel it is better to treat this part as background introduction.

**Materials and Methods**

*Line 94: do you really need to add this information here ?*

**Answer:** Thank you for your careful work. We consider this to be a description of the material so that the reader can better understand our data and we can delete it if necessary

*Line 97: do these information appears in the table?*

**Answer:** Thank you for your careful work. We consider this to be a description of the material so that the reader can better understand our data and we can delete it if necessary

*Line 106: do you really need to add this information?*

**Answer**: Thank you for your careful work. We consider this to be a description of the material so that the reader can better understand our data and we can delete it if necessary

*Line115:* it would be better to add all the other species*?*

**Answer**: Thank you very much for your comments. These comments were also considered by us, but the others group includes many species, mainly species of Myriapoda, Arachnida, Gastropoda (93.98% of the records of others), of which mites, spiders and mollusks account for 72%, and the others species are rarely harmful to WPM, so we did not list all species, but only those represented. We can list if insisted.

*Line 118: I do not think that this table is useful here，If you want, you can add it in the annex*

**Answer**: Your suggestion is greatly appreciated. We have add this table in the annex(Table S1)

**Results and Discussion**

*Line158:* Use the %

**Answer**: Revised accordingly.

*Line305:* Add the %

**Answer**: Revised accordingly.

**Conclusions**

*Line377:* You do not have to repeat the results*?*

**Answer:** Thank you very much for your comments. In our Paper, the purpose is to use results to suggest improvements to inspection and quarantine protocols. Therefore appropriate repetition of results is necessary and we have made partial

deletions to make the paper simpler and clearer
