# Supplementary material for: Quarantine supervision of Wood Packaging Materials (WPM) at Chinese ports of entry from 2003 to 2016
Source: PLoS One. 2021 Aug 5;16(8):e0255762. doi: 10.1371/journal.pone.0255762 (PMC8341634; doi:10.1371/journal.pone.0255762)
Supplement: Attachment — Submitted filename: Reviewer comments.docx [file pone.0255762.s006.docx]

Reviewer’s comments and suggestions

**Introduction,**

***line 51:*** Please, add the Authority of the specie as follows: “*Dendroctonus valens* LeConte and *Bursaphelenchus xylophilus* (Steiner & Bührer) Nickle”

***Line 66:*** Please, change the line as follows: “analysis of Coleoptera in WPM pest interception data from”

***Line 72:*** Please, change the line as follows: “Scolytidae, Cerambycidae, and two other families of insects”

***Line 86:*** Please, put a space after "strategies"

***Line 87:*** 2003 or 2004? In other parts of the paper and in the title there is 2004: please, check and correct

**Materials and Methods**

***Line 118:*** 2003 or 2004?

***Line 122:*** Please, put a space after "records"

***Line 142:*** Please, correct the table by adding a line to separate each category of pests from other. In the current way it is difficult to read correctly. In addition, please, put a space between “cases” and “[16, 28]”

**Results and Discussion**

***Line 151:*** Please, put a space between "officers" and “[29-31]”

***Lines 155-156:*** Please, put a space between "interceptions" and “[32-33]”

***Lines 195-198:*** The Fig. 3 not clearly explains the results: the graph does not show the trend in the four quarters in each year as said in the text

***Line 200:*** Please, put a space between "sectors" and “[35-36]”

***Line 210:*** Please, put a space between "itself" and “[37]”

***Line 253:*** Please, put a space between "underestimated" and “[38]”

***Line 255:*** Table 2: I suggest of enlarge the second column (intitled “Rank order by total interceptios…” ) and fit the text in order to make the table more readible

***Line 273:*** Please, put a space between "categories” and “(Table S2)"

***Line 279:*** Please, remove brachet after “scores”

***Line 280:*** Please, remove "(xylophagous insects)” because is redundant (already said in line 276)

***Line 297:*** Please, put a space between "species" and “[39, 40]”

***Line 300:*** Please, put a space between "damage" and “[41, 42]”

***Line 302:*** Please, put a space between "(regions)" and “[43, 44]”

***Line 305:*** Fig 5: Please, improve the readability of the figure

**Inspection stations**

***Line 314:*** Please, put a space between "that" and “-”

***Line 315:*** Please, put a space between "recorded" and “-”

***Line 330:*** Please, put a space between "provinces" and “[45]”

***Line 346-350:*** In the Fig 6 there are not the abbreviations that are indicated in the caption. Olease, check and correct

***Line 387:*** Please, put a space between "cargo" and “[46]”

**References**

***Line 476:*** Please, change “126+8” in “126-8”

***Line 560:*** Please, change “Sci．. 2013” in “Sci. 2013”
